# Primary Cutaneous Coccidioidomycosis Presenting on the Leg of an Immunocompetent Patient: A Case Report

**DOI:** 10.1155/crdi/5308187

**Published:** 2026-02-26

**Authors:** Alexander Kaminsky, Ikenna Nebo, Sara Suhl, Cynthia M. Magro, Larisa J. Geskin, Lindsey A. Bordone

**Affiliations:** ^1^ Department of Dermatology, Columbia University Vagelos College of Physicians and Surgeons, New York, New York, USA, vagelos.columbia.edu; ^2^ Department of Pathology, Weill Cornell Medicine, New York, New York, USA, cornell.edu; ^3^ Department of Dermatology, Columbia University Irving Medical Center, New York, New York, USA, columbia.edu

**Keywords:** case report, coccidioidomycosis, cutaneous, immunocompetent

## Abstract

Coccidioidomycosis is a fungal infection that primarily manifests as an asymptomatic condition caused by inhalation of fungal spores. Less commonly, patients can develop dissemination to extrapulmonary locations such as the skin or primary cutaneous inoculation of the fungus at a site of trauma. These cutaneous complications are primarily found among immunosuppressed individuals. Here, we present a patient with no relevant past medical history that, after living in an area to which Coccidioides is endemic, developed a slowly growing, pruritic plaque on his right thigh with well‐demarcated areas of hypopigmentation and lichenification with erythematous borders; after several inconclusive biopsies, fungal spherules characteristic of coccidioidomycosis were found on pathology. Along with morphologic evidence of trauma at the site of the infection, the most likely diagnosis was determined to be primary cutaneous coccidioidomycosis. This case is notable for several reasons. First, the patient had a persistent cutaneous coccidioidomycosis infection, with a rare clinical presentation, despite no history of immunocompromising conditions or medications. Second, the patient experienced disease progression while on an empiric trial of a Janus Kinase (JAK) inhibitor. Taken together, these findings may suggest a clinical distinction between the presentation of cutaneous fungal infections in immunocompetent and immunocompromised patients.

## 1. Introduction

Coccidioidomycosis, or “Valley Fever,” is a fungal infection endemic to arid regions of the southwestern United States and parts of Central and South America, most commonly transmitted through the inhalation of airborne spores. While primarily an asymptomatic condition, in rare cases, patients can develop pulmonary complications or dissemination to extrapulmonary locations such as the skin [1]. Rarer still, however, is primary inoculation of the fungus in the skin; this typically presents as an indurated nodule secondary to trauma that resolves on its own within several weeks [2]. Here, we present a patient with no relevant past medical history who, after living in an area to which Coccidioides is endemic, developed a diffuse, long‐standing, slowly growing pruritic plaque on his right anterior thigh that proved difficult to diagnose after several inconclusive biopsies. Histopathologic findings characteristic of coccidioidomycosis were found on a seventh, and final, biopsy.

## 2. Case Presentation

A previously healthy 24‐year‐old year male presented with a slow‐growing, pruritic lesion on his anterior right thigh. It was first noted 2 years prior to presentation, while the patient was living in Arizona, and he reported no known history of trauma to the area or pulmonary symptoms. He subsequently sought care while on a trip to China, and two biopsies of the site were performed. Per the patient, the first was inconclusive and the second contained evidence of fungal elements but was read as nondiagnostic. He had negative HIV testing at this time. He was treated with oral itraconazole (dose unknown) for 6 months and reported improvement but not complete resolution of the rash during this time. Upon cessation of the antifungal therapy, the rash worsened and was accompanied by intermittent pruritus. The patient then completed a course of terbinafine, with partial improvement reported.

**FIGURE 1:**
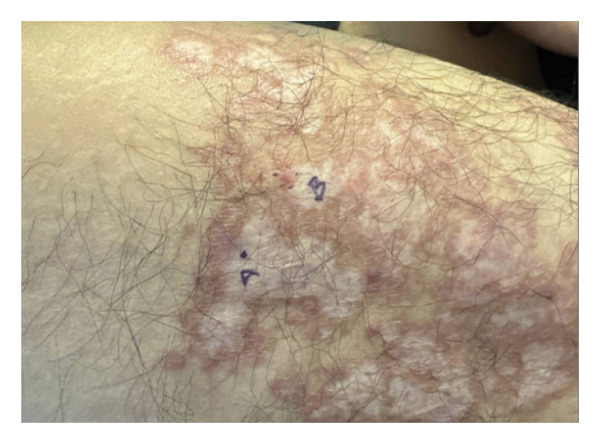
Anterior right thigh approximately 3 years after development of the initial lesion. The final biopsy sites are labeled A and B.

Several weeks after completing the course of terbinafine, he presented to Columbia Dermatology. On evaluation, the patient’s right thigh was found to have a slightly lichenified, scaly, erythematous‐to‐hyperpigmented, well‐demarcated, large plaque with scattered areas of reduced lichenification and hypopigmentation (see Figure 1).

Given this presentation and lack of a definitive diagnosis, the patient underwent two 4.0 mm punch biopsies. Both revealed abundant eosinophilia and poorly formed granulomas that contained multinucleated giant cells. PAS, GMS, AFB, FITE, and spirochete stains were all negative. The samples were sent to the University of Washington for further molecular testing, consisting of fungal and mycobacterial DNA detection by PCR; this ultimately returned negative as well.

Due to the patient’s previous response to antifungal therapy, topical ketoconazole 2% cream was initiated, but subsequent follow‐up revealed minimal improvement. Bloodwork included an unremarkable CBC and liver panel, a negative vasculitis panel (including myeloperoxidase antibody and antineutrophil cytoplasmic antibodies), and a negative QuantiFERON. Karius testing (NGS detection of microbial cell‐free DNA in plasma) was subsequently performed; the patient’s plasma was screened for hundreds of species of bacteria, DNA viruses, fungi, and protozoa, but no microbes were detected at statistically significant levels.

**FIGURE 2:**
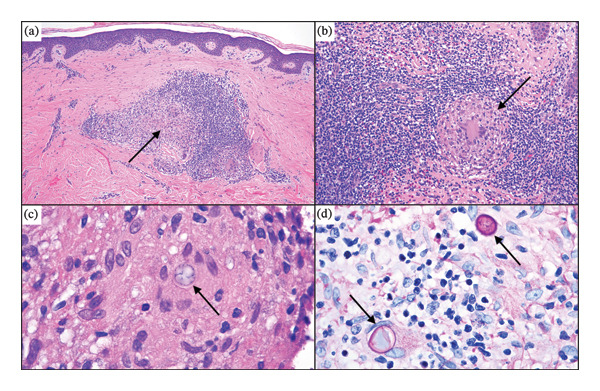
Histopathologic images of the final biopsy. (a) Low power magnification shows a background of cicatricial fibrosis with a supervening, nodular, granulomatous, and lymphocytic infiltrate in the superficial dermis. (b) This higher power image demonstrates the composition of the infiltrate, with many eosinophils, lymphocytes, and epithelioid granulomas (H and E, 200x). (c) A large fungal spore compatible with a mother sporangium is visible within the cytoplasm of a giant cell (H and E, 1000x). (d) PAS staining highlights fungal elements: Two large mother sporangia (fungal spherules) are visible in the center of the granuloma (PASD, 1000x).

At this point, the diagnosis remained unclear. The clinical appearance of the lesions (raised, arciform borders with central clearing) raised suspicion for granuloma annulare. Although histologic findings (poorly formed granulomas accompanied by lymphocytes and eosinophils) were inconsistent with this diagnosis, an off‐label trial of upadacitinib was begun. However, he discontinued this treatment after 3 weeks due to worsening skin involvement and pruritus. Given the treatment‐resistant behavior of the rash, another set of two biopsies was performed 8 months after the patient’s initial presentation, which revealed the findings shown in Figure 2.

**FIGURE 3:**
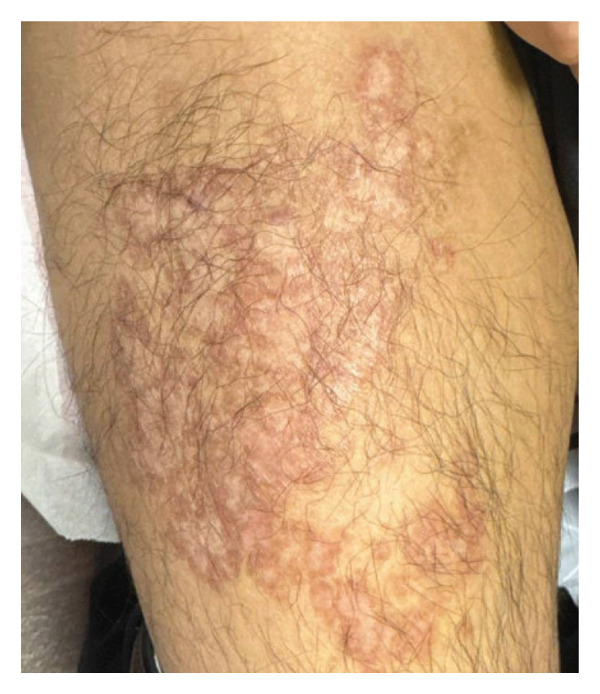
Anterior right thigh after 2 months of treatment with oral itraconazole. The lesion has improved appearance and is no longer pruritic.

Both biopsies showed a florid, benign, eosinophil‐enriched lymphocytic and granulomatous infiltrate (Figure 2(a)) in a background of cicatricial fibrosis compatible with antecedent trauma. The granulomas were cohesive, predominated by epithelioid histiocytes with a few admixed Langhans giant cells and exhibited central fibrinoid degeneration (Figure 2(b)). Some of the granuloma had large, centrally disposed fungal spores in the 20‐micron range (Figure 2(c)); these fungal elements were further highlighted by PAS staining (Figure 2(d)). Although no molecular testing or fungal culture was performed, these clear histologic findings, combined with the patient’s history, strongly suggested a diagnosis of cutaneous coccidioidomycosis. Thus, the patient was started on oral itraconazole 200 mg BID. At follow‐up visits two and 4 months after initiation of this therapy, the patient presented with a large hyperpigmented plaque with internal areas of clearance and hypopigmentation without erythema or pruritus, representing improvement since his prior visits (Figure 3). Further communication with the patient 11 months after initiation of oral antifungal therapy revealed continued symptomatic relief.

## 3. Discussion

Cutaneous manifestations of this fungal infection can have several possible etiologies: reactive cutaneous processes, primary pulmonary disease from spore inhalation with subsequent dissemination to the skin, and primary cutaneous coccidioidomycosis [2]. Of these three, primary cutaneous infection is extremely rare, with several dozen cases reported in total since 1926 [2].

Regarding the pathology of the final biopsy, the differential diagnosis included sarcoidosis; this was disfavored, as lymphoid hyperplasia and tissue eosinophilia would be unusual for this condition. Coccidioidal biopsies, however, consistently display tissue eosinophilia, sometimes corresponding with peripheral blood eosinophilia [3–5]. This degree of tissue eosinophilia is unusual in biopsies procured from other infections, including many fungi [5], suggesting that the eosinophil‐mediated host response may play a role in limiting the coccidioidal disease progression. Thus, the combination of florid eosinophilia, an epithelioid granulomatous response, and large fungal spherules was determined to be most consistent with coccidioidomycosis. No chest X‐ray or coccidioidomycosis serologies were performed; however, given that this patient was otherwise asymptomatic and the pathology indicated a background of cicatricial fibrosis (Figure 2(a)), primary cutaneous coccidioidomycosis secondary to fungal inoculation at a site of trauma was the most likely etiology.

Aside from the condition’s rarity, this case of presumed primary cutaneous coccidioidomycosis (based on histopathology) is notable for several reasons. First, the patient had no history of immunocompromising diseases or medications, a rare finding for a persistent Coccidioides infection. However, a critical clue to the pathogenetic basis of the infection in this patient was morphologic evidence of trauma at the site of the infection. It is established that primary cutaneous Coccidioides infection can occur in immunocompetent hosts in endemic areas if the skin is compromised [6], defining a form of locus minoris resistentiae. However, this case is unique in its clinical presentation; to the best of our knowledge, these clinical findings—with arcuate plaques with raised borders and central hypopigmentation in a discrete area with morphologic evidence of trauma—have never been described previously in a case of primary cutaneous coccidioidomycosis.

The patient was briefly treated with an off‐label trial of upadacitinib, during which he experienced localized worsening of the disease. The relationship between JAK inhibitors and coccidioidomycosis is underexplored, but several studies suggest that the immunosuppressive effects of JAK inhibitors may increase the risk of pulmonary coccidioidomycosis. In a 2020 retrospective analysis of 135 patients in endemic areas treated with ruxolitinib, 5 patients were diagnosed with pulmonary coccidioidomycosis after initiation of JAK inhibitor therapy, despite having no history of the condition [7]. Furthermore, one patient in the cohort with known history of pulmonary coccidioidomycosis developed disseminated disease 2 years after beginning treatment [7]. A small number of case reports have lent additional support to a potentially increased risk in this patient population [8]. Regarding JAK inhibitors and other fungal infections, a more significant body of literature exists. Case reports have linked this class of drugs to primary candidiasis [9], Pneumocystis [10, 11], aspergillosis [12], cryptococcosis [13–16], and histoplasmosis [17]. The link between JAK inhibitors and opportunistic tuberculosis is well‐documented [18], resulting in routine TB testing before initiation of anti‐JAK therapy. Thus, these studies suggest there may be utility in obtaining baseline coccidioidomycosis serologies in patients in endemic areas before starting JAK inhibitors, as well as frequent monitoring for worsening infection.

This case also illustrates a systematic approach to working up a granulomatous infiltrate. After performing a biopsy, the next step is to incorporate special stains for infectious organisms, such as AFB for mycobacteria and GMS or PAS for fungi. Although no molecular testing (tissue PCR) or fungal culture was performed in this case, these can be useful next steps if stains are negative, but clinical suspicion remains. Finally, ancillary testing can be performed based on the differential diagnosis; infectious serologies, a chest X‐ray (for example, in the case of tuberculosis), and autoimmune screening (such as ANCA or ANA) may be indicated. As discussed above, because cutaneous coccidioidomycosis is most often secondary to pulmonary involvement, a chest X‐ray would be a reasonable next step, and was only not performed in this case due to clear morphologic evidence of trauma at the site of infection. Finally, as discussed above, the Karius test is a special molecular serologic test that is rarely performed, but was felt to be indicated in this case due to the lack of positive results after an extensive workup.

It is noteworthy that in our patient, multiple biopsies were performed before a coccidioidal infection was established. It is possible that immunosuppression induced by the JAK inhibitor trial permitted sufficient proliferation of *Coccidioides* to allow detection. In the absence of such immunosuppression, the patient’s immune system may have been able to partially control the infection and reduce the fungal burden below the threshold of detection on biopsy; this may explain why prior biopsies were inconclusive.

Throughout the patient’s treatment course, the differential diagnosis included an autoimmune process, tuberculosis, sarcoidosis, and rubella reactivation. Fungal infection was also considered, given that fungal elements had previously been seen in the biopsy taken in China. However, this potential diagnosis was deprioritized initially on the basis of subsequent nondiagnostic biopsies, the overall clinical presentation, and the fact that the patient was not immunocompromised. This underscores the importance of considering coccidioidomycosis when treating patients from endemic areas; the infection has the ability to mimic many other conditions clinically and complicate efforts at reaching a definitive diagnosis.

## 4. Conclusion

This is a case of a previously healthy 24‐year‐old year male with a long‐standing pruritic lesion on his anterior right thigh that had previously undergone multiple inconclusive biopsies. Ultimately, a diagnosis of primary cutaneous coccidioidomycosis was confirmed histopathologically. This case is notable because of the patient’s unusual clinical presentation, the patient’s immunocompetent status, and the number of inconclusive biopsies required to reach a diagnosis.

## Funding

No funding was received for this research.

## Disclosure

This manuscript has not been posted as a preprint. This work has not been presented at a scientific conference or seminar.

## Ethics Statement

Institutional Review Board approval was not required for this study, as it is a single‐patient case report.

## Consent

Written informed consent for publication of clinical details was obtained from the patient. A copy of the consent form is available upon request.

## Conflicts of Interest

Dr. Larisa J. Geskin has served as an investigator for and/or received research support from Helsinn Group, J&J, Mallinckrodt, Kyowa Kirin, Soligenix, Innate, Merck, BMS, and Stratpharma; on the speakers’ bureau for Helsinn Group and J&J; and on the scientific advisory board for Helsinn Group, J&J, Mallinckrodt, Sanofi, Regeneron, and Kyowa Kirin. The other authors declare no conflicts of interest.

## Data Availability

The data that support the findings of this study are available on request from the corresponding author. The data are not publicly available due to privacy or ethical restrictions.
